# Supplementary material for: The interactome of intact mitochondria by cross-linking mass spectrometry provides evidence for coexisting respiratory supercomplexes
Source: Mol Cell Proteomics. 2017 Dec 8;17(2):216–32. doi: 10.1074/mcp.RA117.000470 (PMC5795388; doi:10.1074/mcp.RA117.000470)
Supplement: Supplemental Data [file supp_RA117.000470_133922_0_supp_23978_fzffwf.zip › spectra_annotation/mito_DR_spectra_annotation/8-1-5-1-6-1.pdf]

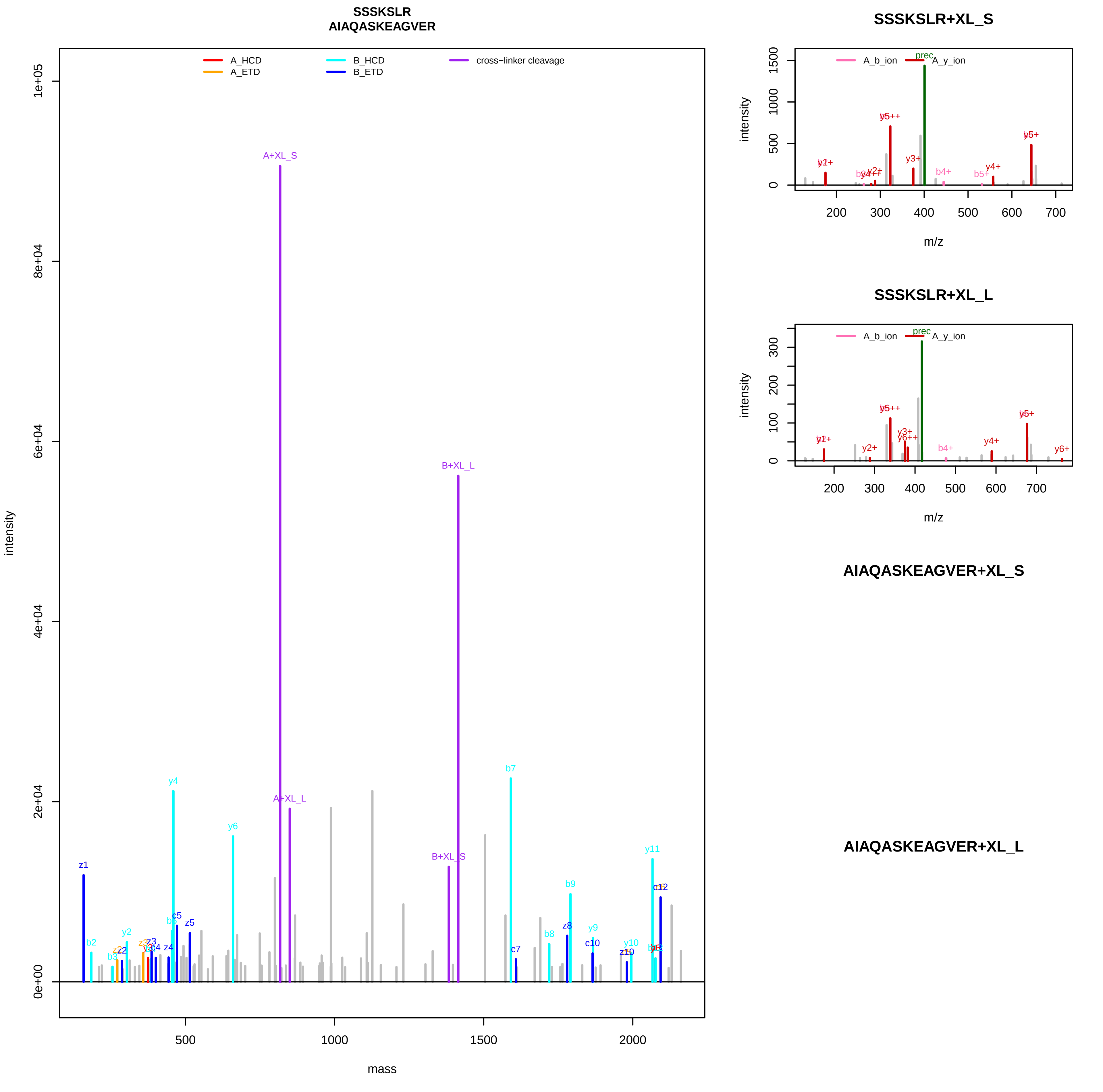
SSSKSLR
AIAQASKEAGVER
A_HCD
A_ETD
B_HCD
B_ETD
cross−linker cleavage
A+XL_S
B+XL_L
A+XL_L
B+XL_S
y4
y6
b7
y11
b9
c12
z1
b2
b3
y2
z2
z3
c4
z4
c5
z5
c7
b8
z8
y9
c10
y10
z10
intensity
mass
0e+00
2e+04
4e+04
6e+04
8e+04
1e+05
500
1000
1500
2000
SSSKSLR+XL_S
A_b_ion
A_y_ion
prec
y5++
y5+
y3+
y1+
y2+
y4+
b4+
b5+
m/z
0
500
1000
1500
200
300
400
500
600
700
SSSKSLR+XL_L
A_b_ion
A_y_ion
prec
y5++
y5+
y3+
y6++
y1+
y2+
b4+
y4+
y6+
m/z
0
100
200
300
200
300
400
500
600
700
AIAQASKEAGVER+XL_S
AIAQASKEAGVER+XL_L
